# The Cause of Death of a Child in the 18th Century Solved by Bone Microbiome Typing Using Laser Microdissection and Next Generation Sequencing

**DOI:** 10.3390/ijms18010109

**Published:** 2017-01-06

**Authors:** Valeria D’Argenio, Marielva Torino, Vincenza Precone, Giorgio Casaburi, Maria Valeria Esposito, Laura Iaffaldano, Umberto Malapelle, Giancarlo Troncone, Iolanda Coto, Paolina Cavalcanti, Gaetano De Rosa, Francesco Salvatore, Lucia Sacchetti

**Affiliations:** 1 CEINGE-Biotecnologie Avanzate, via G. Salvatore 486, 80145 Naples, Italy; dargenio@ceinge.unina.it (V.D.); precone@ceinge.unina.it (V.P.); giorgio.casaburi@gmail.com (G.C.); espositomari@ceinge.unina.it (M.V.E.); iaffaldano@ceinge.unina.it (L.I.); iolanda.coto@unina.it (I.C.); 2 Department of Molecular Medicine and Medical Biotechnologies, University of Naples Federico II, via Pansini 5, 80131 Naples, Italy; 3 Laboratory of Archeo-Anthropology, University of Naples Suor Orsola Benincasa, via Suor Orsola 10, 80125 Naples, Italy; marielvatorino@libero.it; 4 Department of Public Health, University of Naples Federico II, Naples, via Pansini 5, 80131 Naples, Italy; umberto.malapelle@unina.it (U.M.); giancarlo.troncone@unina.it (G.T.); 5 Microbiology Unit, Hospital of Cosenza, via San Martino, 87100 Cosenza, Italy; batteriologia@libero.it; 6 Department of Advanced Biomedical Sciences, University of Naples Federico II, via Pansini 5, 80131 Naples, Italy; gaderosa@unina.it; 7 IRCCS (Istituto di Ricovero e Cura a Carattere Scientifico)-Fondazione SDN, via Gianturco 113, 80143 Naples, Italy

**Keywords:** metagenomics, human microbiome, cold case, next generation sequencing

## Abstract

The history of medicine abounds in cases of mysterious deaths, especially by infectious diseases, which were probably unresolved because of the lack of knowledge and of appropriate technology. The aim of this study was to exploit contemporary technologies to try to identify the cause of death of a young boy who died from a putative “infection” at the end of the 18th century, and for whom an extraordinarily well-preserved minute bone fragment was available. After confirming the nature of the sample, we used laser microdissection to select the most “informative” area to be examined. Tissue genotyping indicated male gender, thereby confirming the notary’s report. 16S ribosomal RNA sequencing showed that *Proteobacteria* and *Actinobacteria* were more abundant than *Firmicutes* and *Bacteroidetes*, and that *Pseudomonas* was the most abundant bacterial genus in the *Pseudomonadaceae* family. These data suggest that the patient most likely died from Pseudomonas osteomyelitis. This case is an example of how new technological approaches, like laser microdissection and next-generation sequencing, can resolve ancient cases of uncertain etiopathology. Lastly, medical samples may contain a wealth of information that may not be accessible until more sophisticated technology becomes available. Therefore, one may envisage the possibility of systematically storing medical samples for evaluation by future generations.

## 1. Introduction

Past cases of death from unknown causes, particularly those of famous personalities, are commonly referred to as “mysterious deaths”. In many instances, such cases can now be solved thanks to advances in research and to the advent of modern technologies [1,2]. Notably, identification of causes of death in ancient “cold cases” may also shed light on the evolution of a disease [3]. This article concerns a young boy who died at the end of the 18th century from a putative infection of a bone wound (possibly osteomyelitis). His parents felt that their son was not receiving appropriate treatment because his condition was not correctly diagnosed. Consequently, they took the unusual step of preserving a fragment of the affected bone for future analysis. Thus, the fragment was sealed by a public notary in a small envelope (see the documentation in the Supplementary File 1 and Figure S1) until the study described in this paper.

Next-generation technology for high-throughput sequencing of nucleic acids has been a breakthrough in the field of metagenomics, and has produced a wealth of data about various environmental niches in medical research, particularly in the field of infectious diseases [4,5]. The aim of our study was to try to identify the cause of death of this boy using contemporary technologies and methods.

## 2. Results

**Figure 1:**
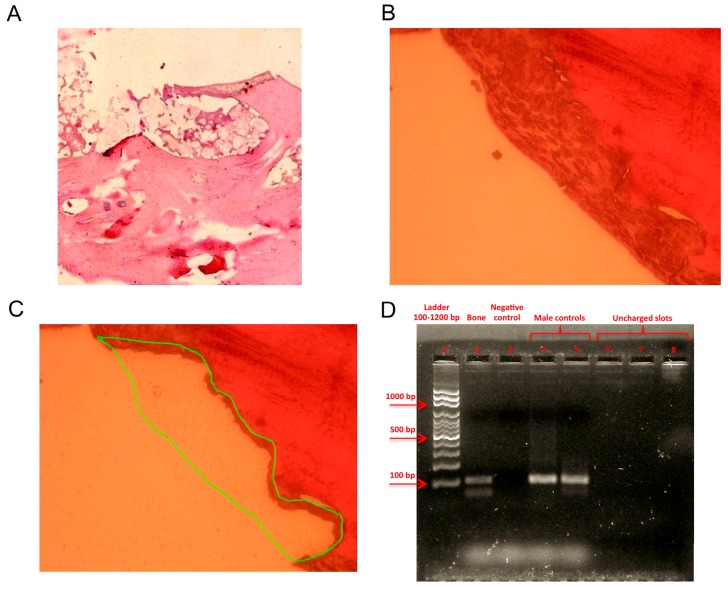
Bone sample characterization. (**A**) Microscopy image (hematoxylin and eosin staining) showing the features typical of bone tissue and inflammatory infiltration; example of a laser captured microdissected area of the bone sample: (**B**) shows the pre-microdissected area (40× resolution); (**C**) the green line encircles the microdissected area; (**D**) sex-determining region Y amplification revealed the sex of the patient: lane 1: ladder (100–1200 bp); lane 2: sample; lane 3: negative control; lanes 4 and 5: male controls; lanes 6–8: uncharged slots.

Hematoxylin and eosin (H and E) stained sections of the specimen showed the features typical of bone tissue and aggregates of granulocytes, which is indicative of an inflammatory reaction (Figure 1A). To minimize the risk of external contamination and obtain a sufficient quantity of DNA-rich material, we isolated a piece of the infected bone by laser microdissection under sterile conditions. Figure 1B shows the pre-microdissected area, and Figure 1C the post-microdissected area. Sex-determining region Y (SRY) analysis confirmed that the DNA was from a male (Figure 1D).

**Figure 2:**
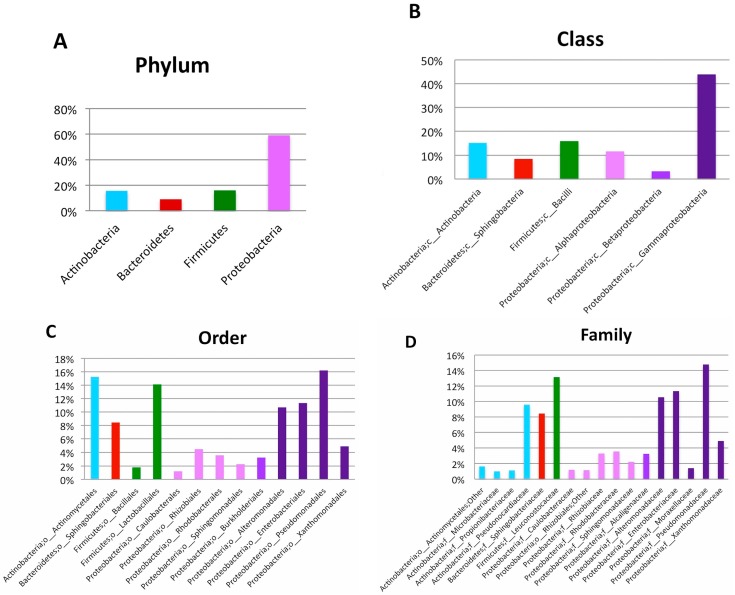

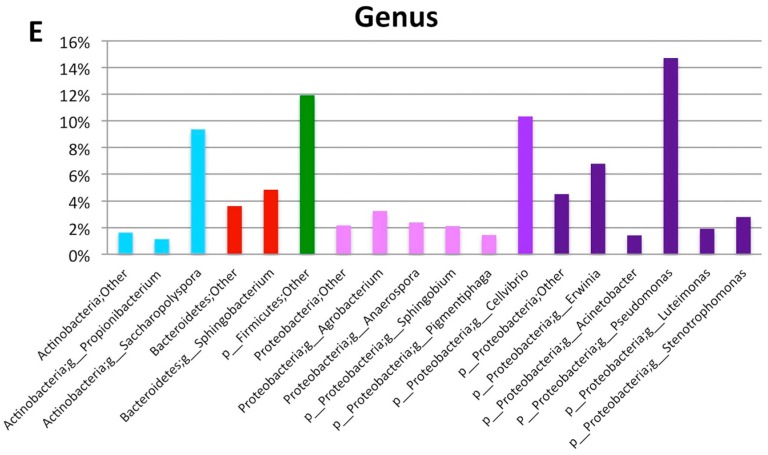
Bone microbiome composition. (**A**–**E**) 16S rRNA sequencing of the bone tissue microbiome. Bacterial composition (frequency > 1%) is shown from phylum to genus level. (**A**) *Proteobacteria* was the most abundant phylum with a frequency of 59%; within this phylum, we found the prevalence of Gammaproteobacteria (45% of frequency, class level, panel **B**); Pseudomonadales (16% of frequency, order level, panel **C**); Pseudomonadaceae (15% of frequency, family level, panel **D**); and *Pseudomonas* (15% of frequency, genus level, panel **E**).

We analyzed the entire bacterial microbiome of the bone sample by a next-generation sequencing approach, as described under Methods (see the Section 4.5). A total of 7,357,569 base pairs (bp) corresponding to 14,172 16S sequences (mean sequence length, 528.7 bp and average quality score 34.18) were generated. After pre-quality filtering (see Section 4.6 for details), 12,829 high quality sequences were considered for subsequent analysis. In detail, 243 representative operational taxonomic units (OTUs) were obtained using an open-reference OTU picking approach [6]. The taxonomic assignment of these OTUs revealed four bacterial phyla. As shown in Figure 2A, the most abundant phylum was Proteobacteria (59%) followed by Actinobacteria and Firmicutes (16% each) and Bacteroidetes (9%). A low percent of OTUs (0.2%) was unclassified (data not shown). The bacterial distribution at the class level (Figure 2B) show the prevalence of Gammaproteobacteria, Bacilli and Actinobacteria (at a frequency of 45%, 16% and 15%, respectively). At Order level (Figure 2C), six orders were most abundant: *Pseudomanadales* (16%), *Actinomycetales* (15%), *Lactobacillales* (14%) *Enterobacteriales* (11%), *Alteromonadales* (10%), and *Sphingobacteriales* (8%). Figure 2D shows the main bacterial families, six of which had more than 1000 assigned sequences: Sphingobacteriaceae (9%), Pseudonocardiaceae (about 10%), Alteromonadaceae (11%), Enterobacteriaceae (12%), Leuconostocaceae (14%), and Pseudomonadaceae (15%). Interestingly, as shown in Figure 2E, Pseudomonadaceae presents the most abundant bacteria at genus level (i.e., *Pseudomonas*; 15%). 

A complete list of the bacteria that reached the deepest taxonomic assignment (i.e., genus) using a filter of 0.05% of OTU frequency is shown in Table 1 and Supplementary Figure S2.

## 3. Discussion

Osteomyelitis has long been known, but the cause of this infection remained obscure until the advent of methods able to identify disease-causing bacterial species. For instance, *P. aeruginosa* was discovered only in 1882. This bacterium is present in diverse chronic severe infectious diseases and is also one of the most difficult to eradicate, a notable case being cystic fibrosis [7,8]. *P. aeruginosa* has been also reported as causative agent of osteomyelitis [9].

We had the unusual opportunity of evaluating with contemporary techniques a well-preserved bone fragment from a child who died in the 18th century from a putative infection. The abundance of the genus *Pseudomonas* and the semi-chronic nature of the infection in the case reported (see Supplementary Materials) strongly suggest that the cause of death was an infection by this bacterial genus. This diagnosis is supported by the whole dataset analyzed. In fact, after assessing the nature of the recovered ancient bone fragment, the analysis of the entire microbiome composition of the bone showed that *Pseudomonas* (within the Pseudomonadaceae family) was the most abundant genus identified (15%), which suggests it was the disease-etiopathogenetic agent of our “cold case” [10].

Environmental contamination cannot be excluded in such an ancient bone sample. However, we detected low abundance of genera typically found in soil or plants (e.g., *Agrobacterium* and *Janthinobacterium*, see Table 1); consequently, we exclude that their presence could affect the result relative to the putative pathogenic role of *Pseudomonas*, which was highly present in our sample. Further, among the most abundant genera, *Pseudomonas*, *Sphingobacterium*, and *Cellvibrio*, only the first one includes species which are highly pathogenic [8,10,11,12]. In line with our hypothesis, *Pseudomonas* was recently identified in five patients affected by diabetic foot osteomyelitis [13]. In the latter study, 16S rRNA sequencing showed that *Pseudomonas* constituted 18% to the total bacterial population, similar to the 15% observed by us.

The rare presence of this lineage in our sample suggests that the possibility of analyzing antique remains of biological samples is certainly useful to be approached by micro-environment microbiome typing. Lastly, the relevant authorities could consider the feasibility of setting up archives of “difficult-to-diagnose” biological material for future evaluation.

## 4. Materials and Methods

### 4.1. Sample Obtainment and Preparation for Analysis

The bone fragment studied was contained in a sealed envelope deposited in the office of a Public Notary on 17 December 1792. On 5 May 1995, after obtaining authorization from the Ministry of Cultural Heritage (authorization number 2.2344), the tiny fragment of bone was extracted from the envelope under sterile conditions. The fragment was trapezoidal in shape, it measured 10 mm × 15 mm, and was white with jagged edges. This information is deposited in an official act of the National State Archives, Cosenza, Italy 5 May 1995. Now, the remaining bone fragment is deposited in the CEINGE-Biotecnologie Avanzate Bio-bank (2nd floor, lab B2/006, fridge block nr. 6). A very minute amount of DNA is still available.

### 4.2. Hematoxylin and Eosin Staining

H and E 5 μm paraffin embedded sections were obtained as previously described [14].

### 4.3. Laser Microdissection and DNA Extraction

Using laser microdissection in combination with a laser pressure catapulting (PALM) system (MicroBeam, Microlaser Technologies GmbH, Bernried, Germany), fixed cells were selectively dissected from 5-μm paraffin-embedded H and E stained slides, under visual control according to the manufacturer’s guidelines. Microdissected cells were collected in the adhesive cap of a 0.5-mL plastic tube (Carl Zeiss, Munich, Germany); 10 μL of tissue lysis buffer A and 2 μL of proteinase K (Qiagen, Crawley, West Sussex, UK) were added to the cells, which were then incubated for four hours at 56 °C. After proteinase K inactivation, the DNA was purified using the QIAamp DNA Mini Kit (Qiagen) following the manufacturer’s instructions. DNA quality was assessed by gel electrophoresis showing a high molecular weight band with a smear, thus ensuring that the DNA quality was sufficient for amplification and sequencing (Supplementary Figure S3A). To exclude external contamination, we also carried out the extraction procedure on the inside of the envelope well separated from the bone fragment, but no DNA was obtained, which indicated that no effective external contamination occurred (Supplementary Figure S3B).

### 4.4. Sex-Determining Region Y Amplification

PCR was carried out with 20 pmol of each of the primers previously used by Shekhtman et al. [15], 1.69 nmol of each dNTP, 1× PCR HotMaster Taq Buffer (5 Prime, Milan, Italy), 0.5 U of HotMaster Taq (5 Prime), and 2 μL of template DNA solution in a total volume of 25 μL. PCR conditions were as follows: initial denaturing at 94 °C for 2 min; 35 cycles of 94 °C for 30 s, 55 °C for 30 s, 65 °C for 30 s; and a final extension at 72 °C for 2 min. Appropriate positive and negative controls were included. The PCR product underwent electrophoresis in agarose gel at 100 V for 30 min.

### 4.5. Microbiome Typing by 16S rRNA Deep Sequencing

A next-generation sequencing-based approach was used to investigate the microbiome of the bone fragment through deep 16S amplicon analysis. In detail, an aliquot of the extracted DNA was used for PCR amplification and sequencing of the bacterial 16S gene. To investigate the bacterial composition of the bone sample, a 548 bp amplicon, spanning from the V4 to V6 variable regions of the 16S rRNA gene was amplified using the 519F (CAGCAGCCGCGGTAATAC) and the 1067R (TGACGACAGCCATGC) primers [16]. Both primers were modified to obtain fusion primers, each one containing at its 5′ end a universal 454 adaptor (adaptor A for the forward primer and adaptor B for the reverse one). The PCR reaction was carried out with 25 µL of H_2_O, 20 µL of 2.5× HotMaster PCR mix (Eppendorf, Hamburg, Germany), 1.5 µL of each primer at 10 µM, and 60 ng of DNA. The amplification was performed on a DNA ENGINE Chassis (Bio-Rad Laboratories, Hercules, CA, USA) under the following conditions: 2 min at 94 °C, 30 cycles of 94 °C for 40 s, 50 °C for 40 s, and 65 °C for 40 s, and a final extension of 70 °C for 7 min. Appropriate positive and negative controls were also amplified. In particular, to exclude possible contaminations, in addition to the bone DNA, we also carried out the 16S PCR amplicon preparation of the empty eluate obtained from the envelope and of a pure water control. Then, we assessed the presence of amplification on a 2% agarose gel stained with bromide ethidium: both the controls gave no amplification products, thus excluding contamination (Supplementary Figure S4). After visualization by gel electrophoresis, the bone PCR product was purified with Ampure magnetic purification beads (Agencourt Biosciences, Beverly, MA, USA), assessed for quality on a Bioanalyzer 2100 (Agilent Technologies, Santa Clara, CA, USA), and quantified using the Quant-it PicoGreen dsDNA kit (Invitrogen, Carlsband, CA, USA). This purified amplicon represents the next generation sequencing library to be sequenced on the 454 Genome Sequencer FLX instrument (Roche, Penzberg, Germany), following manufacturer’s instructions as previously described [17].

### 4.6. Bioinformatics

16S amplicon sequences were analyzed using QIIME tool v. 1.9.1 (http://qiime.org, Flagstaff, AZ, USA), which allows analysis of high-throughput microbial community sequencing data [18,19]. *sff* files were processed to extract FASTA sequences which were quality filtered using default parameters. In detail, sequences with: (i) length outside bounds <200 nt and >1000 nt; (ii) a quality score <25; (iii) ambiguous bases; (iv) primer mismatches; and (v) homopolymer runs in excess of six bases, were removed. Clustering of OTUs was based on 97% similarity using UCLUST tool (http://drive5.com/usearch/manual/uclust_algo.html, London, UK) [20], which was also chosen to assign taxonomy with a cutoff of 90% identity against the 16S rDNA Greengenes database v. 13_08 [21]. Chimeric OTUs were removed using ChimeraSlayer tool (http://microbiomeutil.sourceforge.net/#A_CS, Cambridge, MA, USA) [22].

## 5. Conclusions

Modern techniques, such as next generation sequencing for nucleic acid analysis, and laser microdissection to select the cells to be analyzed from the matrix of the mixed cells/tissues present in the sample, are now being used also to reveal the “cold cases” of ancient remains. These innovative techniques can reveal specific characteristics or the cause of mysterious deaths. In fact, in the case reported herein, a child of the 18th century died after a lengthy bone disease has now been found as affected by a bacterial strain not known at the time, the *Pseudomonas* genus. This paper might have interesting forensic aspects, and may prompt the preservation of samples of uncertain characteristics for future innovative technologies.

## Figures and Tables

**Table 1:** List of all of the bacteria identified at the deepest reachable taxonomic level in the bone sample by 16S rRNA next-generation sequencing. The bacteria are reported according to their relative abundance (at genus level) without considering OTUs that have not been assigned to the genus level. For each of them, the taxonomic assignment at five phylogenetic levels (from phylum to genus), the relative abundances at genus level (the most abundant being 15% for *Pseudomonas*), and the number of high-quality mapping reads are reported at the genus level. Total reads (post-quality OTU classifications) = 4244. Part of the reads (about 11%) are not assigned down to the genus level). Unidentified reads = 0.009%.

Phylum	Class	Order	Family	Genus	Relative Abundance at Genus Level (%)	Number of High Quality Reads at Genus Level
Actinobacteria	Actinobacteria	Actinomycetales	Brevibacteriaceae	*Brevibacterium*	0.31	9
Actinobacteria	Actinobacteria	Actinomycetales	Corynebacteriaceae	*Corynebacterium*	0.75	22
Actinobacteria	Actinobacteria	Actinomycetales	Microbacteriaceae	*Agrococcus*	0.38	11
Actinobacteria	Actinobacteria	Actinomycetales	Microbacteriaceae	*Leucobacter*	0.10	3
Actinobacteria	Actinobacteria	Actinomycetales	Microbacteriaceae	*Microbacterium*	0.68	20
Actinobacteria	Actinobacteria	Actinomycetales	Micrococcaceae	*Arthrobacter*	0.21	6
Actinobacteria	Actinobacteria	Actinomycetales	Micrococcaceae	*Micrococcus*	0.96	28
Actinobacteria	Actinobacteria	Actinomycetales	Propionibacteriaceae	*Propionibacterium*	1.34	39
Actinobacteria	Actinobacteria	Actinomycetales	Pseudonocardiaceae	*Amycolatopsis*	5.14	150
Actinobacteria	Actinobacteria	Actinomycetales	Pseudonocardiaceae	*Pseudonocardia*	1.13	33
Actinobacteria	Actinobacteria	Actinomycetales	Pseudonocardiaceae	*Saccharopolyspora*	8.87	259
Bacteroidetes	Flavobacteriia	Flavobacteriales	Flavobacteriaceae	*Flavobacterium*	0.41	12
Bacteroidetes	Sphingobacteriia	Sphingobacteriales	Sphingobacteriaceae	*Sphingobacterium*	11.26	329
Firmicutes	Bacilli	Bacillales	Bacillaceae	*Bacillus*	0.72	21
Firmicutes	Bacilli	Bacillales	Planococcaceae	*Lysinibacillus*	0.14	4
Firmicutes	Bacilli	Bacillales	Planococcaceae	*Rummeliibacillus*	0.79	23
Firmicutes	Bacilli	Bacillales	Staphylococcaceae	*Staphylococcus*	0.45	13
Firmicutes	Bacilli	Lactobacillales	Aerococcaceae	*Aerococcus*	0.79	23
Firmicutes	Bacilli	Lactobacillales	Enterococcaceae	*Enterococcus*	0.07	2
Firmicutes	Bacilli	Lactobacillales	Streptococcaceae	*Streptococcus*	0.45	13
Proteobacteria	Alphaproteobacteria	Caulobacterales	Caulobacteraceae	*Brevundimonas*	0.10	3
Proteobacteria	Alphaproteobacteria	Rhizobiales	Rhizobiaceae	*Agrobacterium*	4.86	142
Proteobacteria	Alphaproteobacteria	Rhodobacterales	Rhodobacteraceae	*Anaerospora*	3.87	113
Proteobacteria	Alphaproteobacteria	Rhodobacterales	Rhodobacteraceae	*Rhodobacter*	1.10	32
Proteobacteria	Alphaproteobacteria	Sphingomonadales	Sphingomonadaceae	*Sphingobium*	3.22	94
Proteobacteria	Betaproteobacteria	Burkholderiales	Alcaligenaceae	*Achromobacter*	0.27	8
Proteobacteria	Betaproteobacteria	Burkholderiales	Alcaligenaceae	*Pigmentiphaga*	1.64	48
Proteobacteria	Betaproteobacteria	Burkholderiales	Oxalobacteraceae	*Janthinobacterium*	0.10	3
Proteobacteria	Gammaproteobacteria	Alteromonadales	Alteromonadaceae	*Cellvibrio*	10.50	482
Proteobacteria	Gammaproteobacteria	Enterobacteriales	Enterobacteriaceae	*Erwinia*	4.59	134
Proteobacteria	Gammaproteobacteria	Pseudomonadales	Moraxellaceae	*Acinetobacter*	1.92	56
Proteobacteria	Gammaproteobacteria	Pseudomonadales	Pseudomonadaceae	*Pseudomonas*	15.00	584
Proteobacteria	Gammaproteobacteria	Xanthomonadales	Xanthomonadaceae	*Luteimonas*	2.94	86
Proteobacteria	Gammaproteobacteria	Xanthomonadales	Xanthomonadaceae	*Stenotrophomonas*	3.97	116

## Supplementary Materials

Supplementary materials can be found at www.mdpi.com/1422-0067/18/1/109/s1.

## Abbreviations

H&E	Hematoxylin and eosin
SRY	Sex-determining region Y
OTUs	Operational taxonomic units
