# Supplementary material for: The Cause of Death of a Child in the 18th Century Solved by Bone Microbiome Typing Using Laser Microdissection and Next Generation Sequencing
Source: Int J Mol Sci. 2017 Jan 6;18(1):109. doi: 10.3390/ijms18010109 (PMC5297743; doi:10.3390/ijms18010109)
Supplement: Supplementary file 1 [file ijms-18-00109-s001.zip › ijms-161108-Supplementary Materials/ijms-161108-supplementary figures.pdf]

Supplementary Materials: The Cause of Death of a Child in the 18th Century Solved by Bone Microbiome Typing Using Laser Microdissection and Next Generation Sequencing

Valeria D'Argenio, Marielva Torino, Vincenza Precone, Giorgio Casaburi, Maria Valeria Esposito, Laura Iaffaldano, Umberto Malapelle, Giancarlo Troncone, Iolanda Coto, Paolina Cavalcanti, Gaetano De Rosa, Francesco Salvatore and Lucia Sacchetti

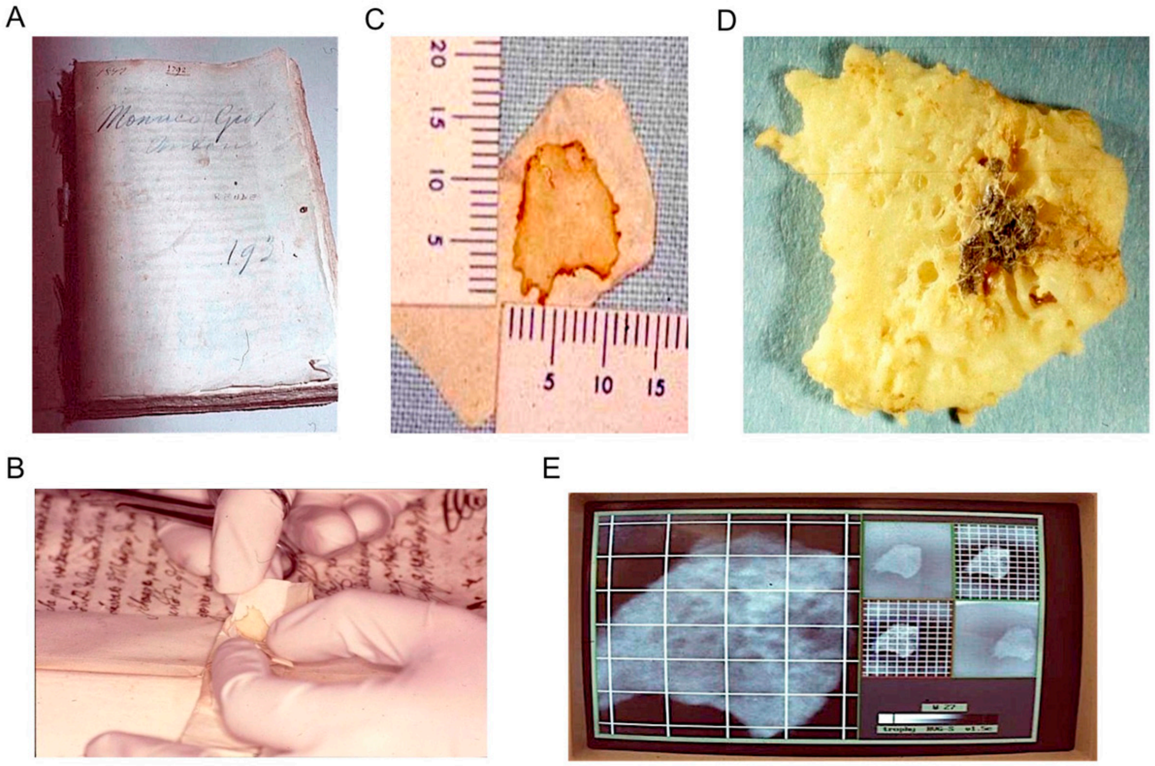

Figure S1. Recovery of bone fragment: (**A**) Notary act of 1792; (**B**) Envelope containing bone opening; (**C–E**) Size of the bone fragment (mm) and bone area identified for microdissection.

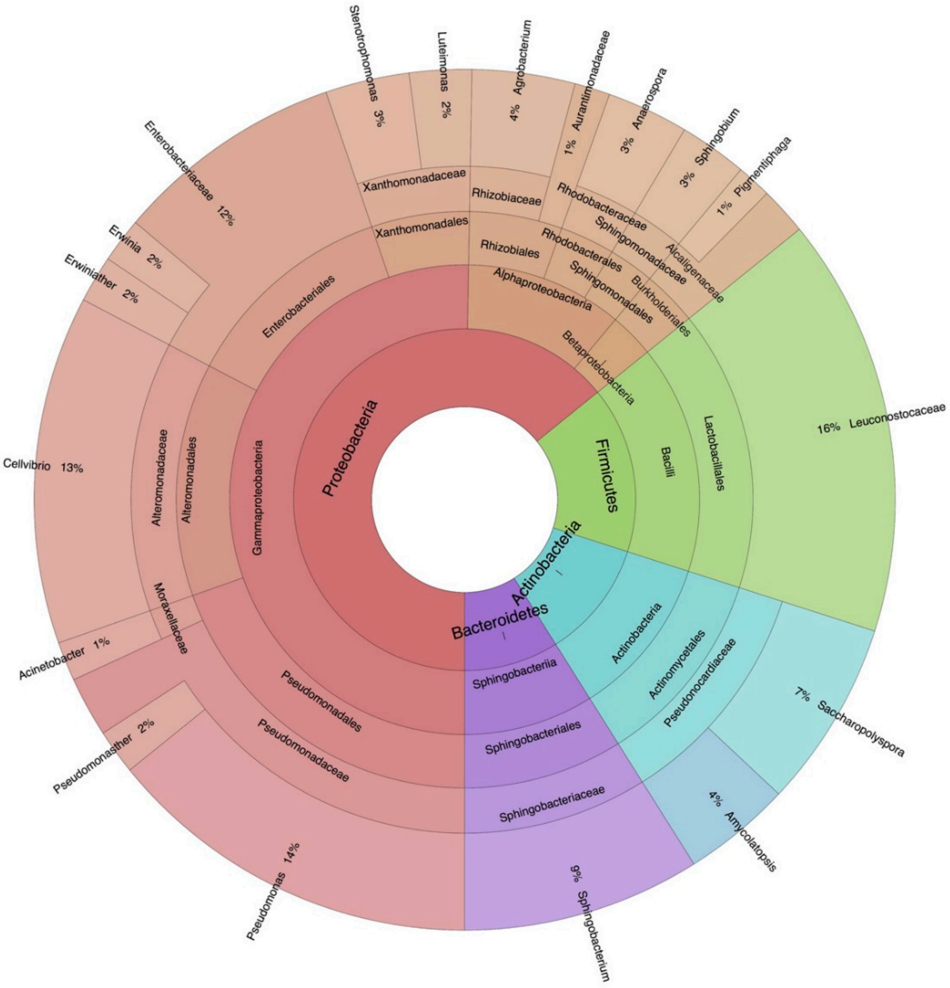

Figure S2. Pie chart of bacteria present in the bone sample with a relative abundance at genus level >1%. The classification "others", indicates all the taxa with a relative abundance <1%.

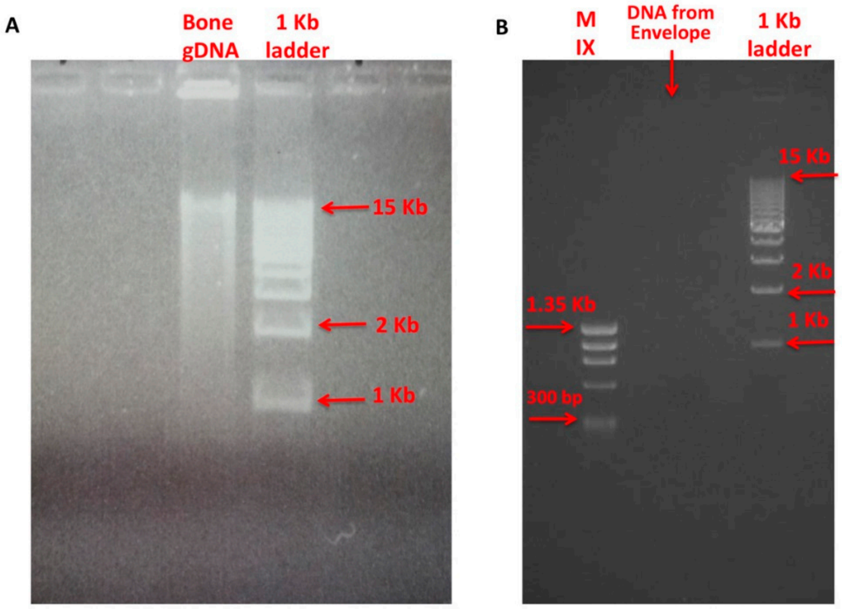

Figure S3. (**A**) Quality evaluation of genomic DNA from the bone on 0.7% agarose gel electrophoresis followed by ethidium bromide staining; (**B**) No DNA was obtained from the envelope that contained the bone (central slot).

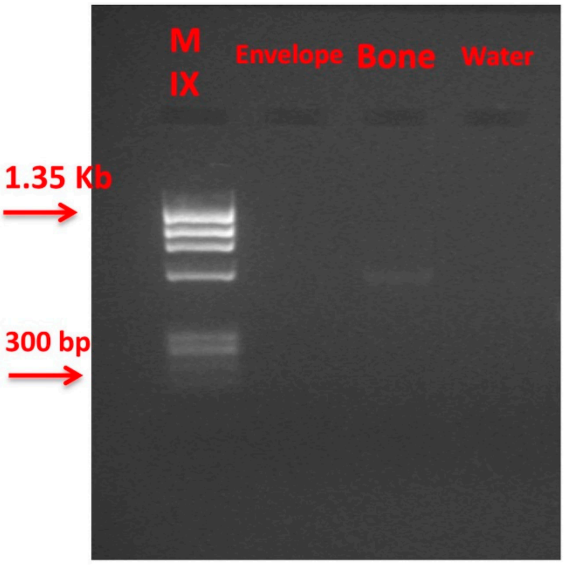

Figure S4. 16S PCR amplicon evaluation on 2% agarose gel stained with bromide ethidium showed no amplification in both the empty eluate obtained from the envelope and the pure water control, thus excluding the presence of contaminants; a faint band, at the level of about 600 bp, is seen in the "Bone" slot only.
