# Supplementary material for: The Cause of Death of a Child in the 18th Century Solved by Bone Microbiome Typing Using Laser Microdissection and Next Generation Sequencing
Source: Int J Mol Sci. 2017 Jan 6;18(1):109. doi: 10.3390/ijms18010109 (PMC5297743; doi:10.3390/ijms18010109)
Supplement: Supplementary file 1 [file ijms-18-00109-s001.zip › ijms-161108-Supplementary Materials/Supplementary File 1.pdf]

A brief hystory of the case report: the case of Vincenzo Maria di Bartolo: a case of osteomyelitis dating from 1792

Reported by: Marielva Torino, University of Naples Suor Orsola Benincasa, Naples, Italy

In a notary act of 1792, received by Notary Giovanni Antio Monaco di Rende (Cosenza), among papers 177 v.[1] and 178 r.[2] there was a fragment of human bone sampled on the night of 15 December 1792.

A careful reading showed that the act was requested by Cinzia Caputo, mother of the youth Vincenzo Maria di Bartolo who was affected by a long-standing sore from which bone fragments emerged despite treatment prescribed by various doctors.

The notary act was contextual to another curettage of the wound performed for treatment purposes at the end of which a bone fragment was obtained that was placed in a small package measuring 2 x 5 cm attached to the notary act and closed with a wax seal of Notary Monaco.

The presence of numerous witnesses, and among these of another notary[3], was due to the particular delicacy of the contents of the act that were not juridical, but concerned the state of health of an individual, which is totally unusual in a notary act and in many respects more problematic as regards the aim of being proof for future reference.

The study of the bone fragment and the discovery of documents at the Parochial Archive of the Church of Santa Maria Maggiore (Rende) and the National Archive (Cosenza) revealed both the reasons why, to fix the date of the boy's disease using the most formal means foreseen by the law, then and today, as well as the entire matter and in particular the microbiological causes of the disease and the boy's death.

****

Rende, 1792 December 15 for indiction (hora vigesima teria)

*For instance and request made to Us National Contract Judges [*National Notary *Board], and witnesses Cintia Caputo, wife of R. Ing. Raffaele di Bartulo of Rende, we convened in their house in Rende called "Sopra la Crocevia" and in the room of her son Vincenzo Maria di Bartulo with the surgeon D. Michele Sgangone, D*[r] *Physicist D. Michele Pittò, Not. Francescantonio Mazziotta, Mr D. Domenico Vanni and Physicist and Surgeon D. Antonio Rovella. And lighted three candles and locked the door and windows, the said surgeon Sgangone uncovered the said sore of the said Vincenzo Maria, and showed to the said Physicist Pittò and Not. Mazziotta that a bone fragment of Vincenzo Maria was the same that was seen the previous evening together with Dr Physicist D. Antonio Glari, absent in this act, which they confirmed and witnessed; and having also the said Dr. Physicist Rovella assured that the said Mr Vanni observed the said bone during the treatment of the surgeon Don Felice Passaresi,*

[1] *verso*
[2] *recto*
[3] Francescantonio Mazziotta

the said Don Domenico suddenly responded that the truth was that several times he was present when Don Felice treated the said sore and that he removed parts of the bone, of which several fragments were present, but he was not able now to distinguish between bone fragments.

Therefore the surgeon Sgangone, with competence, removed the said bone, which was as small as the auricular finger[4]*, and gave it to me as Notary to seal and conserve.*[5]

§§§§§§§§§§§§§§§§§§§§§§

From this document it appears that the notary act was wanted by the boy's mother, who, despite doctors' treatments, saw that her son's sore did not heal. The sore, from which there was a continuous outflow of bone fragments, was probably caused by an old injury, and for that reason it was defined *said* in the act.

It is clear that the mother no longer trusted what the physicians said and believed that the bone fragments were always the same: this explains why, during further medical treatment and curettage of the sore, she asked to seal the extracted bone fragment, as in the attached document.

There is no indication of the age or the date of birth of the young Vincenzo Maria, but this was common in notary acts of that period.

To obtain more information, research was conducted among the notary acts of that time, particularly in those of the notary Monaco of Rende, who was clearly the fiduciary of the di Bartolo family, and in those of the other notary Francescantonio Mazziotta, mentioned as witness in the act, whose presence witnesses the great importance attributed to the intervention. Thanks to the indications found, the research focused on the parochial archive of the Mother Church of Rende and on the State archive of Cosenza.

Vincenzo Maria di Bartolo was the son of Cinzia Caputo and of her second husband[6] the royal engineer Raffaele di Bartolo from Rende. Besides Vincenzo Maria, Mrs Caputo had three daughters with Mr di Bartolo, Maria Isabella, Maria Emanuela, and Maria Rosaria; Mrs Caputo from her first wedding had a daughter named Francesca, as evinced from the testamentary acts of Mrs Caputo and her husband, both drafted on January 22, 1797 by the above-mentioned notary Giovanni Antonio Monaco. At the time the couple lived in the house where the act concerning Vincenzo Maria was drafted, in the district of Piazza sopra la Crocevia, and they dictated their last will on the same day, while in bed, sick in the body but healthy in mind. The mother left the rightful maternal quote to Francesca, daughter of her first marriage. Both parents confirmed the dowry already given on the occasion of their wedding to Maria Rosaria and Maria Emanuela, and they appointed the unmarried daughter Maria Isabella as their universal heir. This act contains no mention of Vincenzo Maria, thus at that time he was already dead[7],

---

[4] The bone was as little as the little finger, namely the finger used to put in the ear

[5] State archive of Cosenza. 498. Acts of the Notary Giovanni Antonio Monaco from Rende. (Cosenza). 1792

[6] Cinzia Caputo, as per the act of the notary Saverio Mazziotta di Rende (Cosenza), dated 10 november 1756, widow of Tommaso Filippello, was married to Raffaele di Bartulo from some months. The notary act was about an agreement drawn up between the woman and her brothers for hereditary matters. In the same act it is written that for the second wedding no matrimonial chapters were drafted.

[7] Cinzia Caputo and Raffaele di Bartolo overcame the disease that pushed them to draft their testament: as emerged from the inventory of the goods of M.Isabella di Bartolo's, drafted on May 4, 1802. Raffaele di Bartulo died on December 31, 1801,

even in the will of his sister Maria Rosaria, drafted by notary Monaco on November 26, 1797 there is no mention of Vincenzo Maria.[8]

Instead, the boy is mentioned in the matrimonial act of his sister Maria Rosaria, drafted on January 30, 1793, that is approximately one month after the act regarding him. The illness of the boy is mentioned in one of the last provisions of the matrimonial act. As a consequence, the parents specified that, in case their daughter Maria Rosaria died without children or in case her children should died at a young age, all the promised dowry should be returned to them and their son Vincenzo Maria. This provision in support of Vincenzo Maria confirms that the disease was still in progress, so that the son was preferred over his sisters. It is interesting to note that Maria Rosaria's betrothed was the Physicist and Surgeon D. Antonio Rovella, one of the witnesses present during the curettage of the sore of Vincenzo Maria, and that, as husband of the woman, he was mentioned in the testament of the mother-in-law, Cinzia Caputo, dated January 22, 1797.[9]

Thanks to the indications emerging from the documents and to the verification that Vincenzo Maria disappears from acts in January 1793, research focused on the Parochial Archive of the Church of Santa Maria Maggiore (Rende) where the birth and date certificates have been found. From these acts it emerges that the boy was born on April 8, 1767[10] and died 18 months after the notary act, on June 26, 1795[11]at the age of 28, probably because of this chronic infection, as we will demonstrate.

****

Thanks to the collaboration of the State archive of Cosenza and of the Paleopathology laboratory of Pisa, it was possible to study the finding; after obtaining the authorization from the Ministry of Cultural Heritage and in the presence of the notary Loredana Grimaldi on May 5, 1995 in Naples[12], once confirmed the integrity of the envelop, it was opened.

A thin fragment of bone was found inside the envelop and it was delicately extracted with sterilized tools. The fragment had a trapezoidal shape, it measured 10x15 mm, and it was white with jagged edges. The macroscopic, radiologic and optic microscopic exam confirmed that it was a bone fragment, probably derived from a long bone.[13]

---

while, at that time Cinzia Caputo was still alive (State archive of Cosenza. 408. Acts of the Notary Giovanni Antonio Monaco from Rende. 1802. 56 v). Cinzia Caputo died before her daughter Maria Isabella, as indicated in the death certificate of Maria Isabella, who died at the age of 36 on October 1, 1814 and at that date, both parents were dead (State archive of Cosenza. 498. Acts of the Notary Giovanni Antonio Monaco from Rende. 1797. 168 v).

[8] Maria Rosaria di Bartolo "sick in the body but healthy in the mind" in the testament appointed her parents as heirs, giving them the two-thirds of her dowry;; the remaining third was given to her spouse, Dr Antonio Rovella (State archive of Cosenza. 498. Acts of the Notary Giovanni Antonio Monaco from Rende. 1797. 168 v

[9] State archive of Cosenza. 408. Acts of the Notary Giovanni Antonio Monaco from Rende. 1797. 23 v

[10] Parochial Archive of the Church of Santa Maria Maggiore (Rende), Baptismal book from 1763 to 1772, 1st Ministry, folio 49 no. 235/bis (posthumously numbering, ed)

[11] Parochial Archive of the Church of Santa Maria Maggiore (Rende), Death book from 1772 to 1801, f.102 recto, act no. 809 posthumously numbering, ed. from the document, it emerges that the boy died after the sacraments.

[12] Act of the notary Grimaldi, 5 May 1995. Rep. No. 7304. Collection no. 982

[13] Unfortunately in the act the location of the *said sore* is not specified, ed.

The nature of the finding and the reading of the act of the notary Monaco suggest that the young Vincenzo Maria was affected by a severe case of chronic osteomyelitis, and that the fragment is a bone sequestration from the infected sore [1].

The healing of Vincenzo might have occurred only after an antibiotic therapy, that was unknown at that time, and then after the surgical removal of the bone sequestration, that has been performed several times on the boy.

Without these combined actions, the disease, especially severe in young people, can cause death.

Osteomyelitis is generally originated by an external infection, because of hematogenously propagation of inflammatory processes of soft parts. The acute hematogen form is frequent in the juvenile age: in the 97% of the cases it appears before 25 ages. Osteomyelitis is often caused by bacteria, especially by *Staphylococus aureus;* occasionally it is caused by *Proteus mirabilis*, *Streptococcus*, *Pneumococcus*, *Pseudomonas*, *Aerobacter aerogenes* or *Hemophylus influenzae* [2, 3].

The initial phase of the infection is characterized by an intense hyperemia of the medullary portion of the cancellous bone, followed by the purulent phase, when the purulent and infected material subcutaneously arrives in the periosteum and in the muscle, until reaching the skin. The involvement of the other bone portions results in osteomyelitis and periostitis [4].

The attempt of the periosteum to recreate the damaged structure and the activity of the osteoclasts to remove the necrotic bone, as well as the low virulence of the infection and the inadequate host response, result in the transformation of the osteomyelitis from acute to chronic. The latter is characterized by the development of bone sequestrations that, being considered as foreign bodies by the organism, are expelled from the painful sore several times [4], as it happened to Vincenzo Maria di Bartolo.
In the chronic form, remission and exacerbation periods lasting months or years can occur [3], resulting in the destruction of wide portions of bone, with the risk of a further dissemination of the infection in bone sites, even far from the primitive location [5].
Signs of hematogen osteomyelitis in children can be found after months or years with intermittent or continuous presence of fistulas from the damaged bones, usually the long bones of the extremities, such as femur, tibia, or humerus with infection of the soft tissue overlying [2]. In the 80% of cases osteomyelitis involves only one bone [6].

The healing can occur only after the surgical removal of the bone sequestration and after antibiotic therapy. Without these combined actions the disease can cause death because of pyaemia and/or toxaemia [7].

Before the advent of antibiotics the mortality was in 25% of the cases because of bacteremia, The use of antibiotics has drastically decreased the frequency of this pathology [6].

The microscopic exam of the bone fragment showed the bone gaps caused by the inflammation covered by fibrous tissue; this result confirms that the organism tried to react to the disease process and to set limits to it.

§§§§§§§§§§§§§§§§§§§§§§§

The researches in the State archive of Cosenza and in the Parochial Archive of the Church of Santa Maria Maggiore (Rende) have been conducted by Dr Lucia Chinigò from the State archive of Cosenza.

Acknowledgements

We thank:
The director of the State archive of Cosenza, Dr Anna Maria Letizia Fazio
The priest of the Church of Santa Maria Maggiore (Rende), Don Domenico Sturino and Mr Lucio Provenzano

In memory of Dr. Vittoria Quarta Cerulo (1943-2013), because thanks to her love for research, when she was director of the State archive of Cosenza the authorization on the investigation on the finding has been obtained.

REFERENCES

1. Torino M, Fornaciari G. Un singolare caso archivistico di osteomielite del 1792. Bollettino di paleopatologia. 1997; 1: 117-120.
2. Harrison's. 1983. *Principles of internal medicine.* 10th Ed. Intemational Student. Ed
3. Aufderheide, A.; Rodriguez-Martin, C. 2005. *The Cambridge Encyclopedia of Human Palopathology.* Cambridge University Press
4. Lanza, G. 1978. *Anatomia patologica sistematica.* Ed. Piccin Padova
5. Robbins S.L.; A. Marcia. 1975 *Principi di patologia.* Ed. Piccin Padova
6. Ortner, D.J.; Putschar, W.G.J. 1981 *Identification oj pathological conditions in human skeletal remains.* Smithsonian Institution Presso Washington.
7. Walter-Israel. 1991. *Patologia generale.* Ed. Ambrosiana
